# 610. Low Rates of Acute Kidney Injury (AKI) in Outpatient Vancomycin Managed by Pharmacists using Precision Dosing Software

**DOI:** 10.1093/ofid/ofac492.662

**Published:** 2022-12-15

**Authors:** Claudia P Schroeder, Kimberly A Couch, Joyce F Gee, Lucinda J Van Anglen

**Affiliations:** Healix Infusion Therapy, LLC, Sugar Land, Texas; Healix Infusion Therapy, LLC, Sugar Land, Texas; Healix Infusion Therapy, LLC, Sugar Land, Texas; Healix Infusion Therapy, LLC, Sugar Land, Texas

## Abstract

**Background:**

AKI is a complication of intravenous vancomycin (VAN) occurring in up to 43% of patients (pts) during hospitalization. Data on AKI in pts receiving VAN through outpatient parenteral antimicrobial therapy (OPAT) are limited. There is suggestion that VAN managed through precision dosing pharmacokinetic (PK) software may limit this occurrence. We evaluated the AKI rate of pts receiving VAN as OPAT through Physician Office Infusion Centers (POICs) over a 12-month period.

**Methods:**

A review was conducted of OPAT VAN pts treated in 2021 with therapeutic drug monitoring (TDM) performed (InsightRX) and ≥2 VAN serum levels. These pts were then evaluated for AKI, defined as increase in serum creatinine (SCr) ≥1.5 times baseline. Pts on dialysis were excluded. Data included comorbidities, diagnosis, VAN regimen, concomitant medications, SCr, and VAN serum levels. PK parameters were collected with trough values and estimated 24-hr area under the curve (AUC_24_). Outcome of therapy was assessed following identification of AKI.

**Results:**

A total of 330 pts from 51 POICs had TDM performed. Of these, 27 (8.2%) developed AKI. Median age was 61 years, 67% were male, and baseline SCr was 0.8 mg/dL (IQR, 0.7-1.0). Most pts (89%) were on concomitant nephrotoxic medications. Median duration of OPAT was 34 days (IQR, 18-41) with onset of AKI after 16 days (IQR, 10-21). At time of AKI, the median SCr increase was 1.7 times baseline (IQR, 1.5-2.0) with a corresponding VAN trough of 21 mg/mL (IQR, 15-28) and AUC_24_ of 572 mg*h/L (IQR, 495-660). As a result, VAN was discontinued in 14 (52%), dose or frequency modified in 10 (37%) with completion of VAN, and no modifications performed in 3 (11%). One pt who discontinued VAN required hospitalization due to AKI with resolution.

**Conclusion:**

VAN managed by pharmacists in an OPAT setting using precision dosing software resulted in a low rate of AKI. Median rise in SCr was < 2 times baseline for those with AKI. Rapid identification of early AKI by the pharmacist resulted in changes to the regimen or VAN discontinuation, preventing serious patient sequelae. This supports the need for pharmacist-led monitoring in an outpatient setting with long VAN therapy durations.

**Disclosures:**

**Lucinda J. Van Anglen, PharmD**, Merck & Co.: Grant/Research Support|Paratek: Grant/Research Support.

